# Tomato SlERF.A1, SlERF.B4, SlERF.C3 and SlERF.A3, Members of B3 Group of ERF Family, Are Required for Resistance to *Botrytis cinerea*

**DOI:** 10.3389/fpls.2016.01964

**Published:** 2016-12-27

**Authors:** Zhigang Ouyang, Shixia Liu, Lihong Huang, Yongbo Hong, Xiaohui Li, Lei Huang, Yafen Zhang, Huijuan Zhang, Dayong Li, Fengming Song

**Affiliations:** ^1^National Key Laboratory for Rice Biology, Institute of Biotechnology, Zhejiang UniversityHangzhou, China; ^2^National Navel Orange Engineering Research Center, College of Life and Environmental Sciences, Gannan Normal UniversityGanzhou, China

**Keywords:** tomato (*Solanum lycopersicum*), ethylene-responsive factor (ERF), B3 group, *Botrytis cinerea*, defense response

## Abstract

The Ethylene-Responsive Factors (ERFs) comprise a large family of transcriptional factors that play critical roles in plant immunity. Gray mold disease caused by *Botrytis cinerea*, a typical necrotrophic fungal pathogen, is the serious disease that threatens tomato production worldwide. However, littler is known about the molecular mechanism regulating the immunity to *B. cinerea* in tomato. In the present study, virus-induced gene silencing (VIGS)-based functional analyses of 18 members of B3 group (also called Group IX) in tomato ERF family were performed to identify putative ERFs that are involved in disease resistance against *B. cinerea*. VIGS-based silencing of either *SlERF.B1* or *SlERF.C2* had lethal effect while silencing of *SlERF.A3* (*Pit4*) significantly suppressed vegetative growth of tomato plants. Importantly, silencing of *SlERF.A1, SlERF.A3, SlERF.B4*, or *SlERF.C3* resulted in increased susceptibility to *B. cinerea*, attenuated the *B. cinerea*-induced expression of jasmonic acid/ethylene-mediated signaling responsive defense genes and promoted the *B. cinerea*-induced H_2_O_2_ accumulation. However, silencing of *SlERF.A3* also decreased the resistance against *Pseudomonas syringae* pv. *tomato* (*Pst*) DC3000 but silencing of *SlERF.A1, SlERF.B4* or *SlERF.C3* did not affect the resistance to this bacterial pathogen. Expression of *SlERF.A1, SlERF.A3, SlERF.B4*, or *SlERF.C3* was induced by *B. cinerea* and by defense signaling hormones such as salicylic acid, methyl jasmonate, and 1-aminocyclopropane-1-carboxylic acid (an ethylene precursor). SlERF.A1, SlERF.B4, SlERF.C3, and SlERF.A3 proteins were found to localize in nucleus of cells and possess transactivation activity in yeasts. These data suggest that SlERF.A1, SlERF.B4, and SlERF.C3, three previously uncharacterized ERFs in B3 group, and SlERF.A3, a previously identified ERF with function in immunity to *Pst* DC3000, play important roles in resistance against *B. cinerea* in tomato.

## Introduction

During last decade, extensive genetics and molecular studies revealed that higher plants have evolved to possess a sophisticated innate immunity system, which is similar to the innate immunity in animals (Dodds and Rathjen, 2010; Fu and Dong, 2013). The innate immunity system in plants consists of two layers of immune responses, called pathogen-associated molecular pattern (PAMP)-triggered immunity (PTI) and effector-triggered immunity (ETI), which are precisely regulated upon infection from different types of pathogens (Bernoux et al., 2011; Segonzac and Zipfel, 2011). Generally, both of PTI and ETI are required for resistance to biotrophic and hemibiotrophic pathogens while only PTI is believed to be active resistance response against necrotrophic pathogens (Mengiste, 2012). Upon sensing and recognition of the pathogen-derived PAMPs or effectors by surface/extracellular receptors such as receptor-like kinases and receptor-like proteins in plants (Osakabe et al., 2013), initiation of the innate immune response often requires transcriptional reprogramming to coordinately regulate expression of a large set of genes (Moore et al., 2011; Buscaill and Rivas, 2014). For example, approximately one-third of the Arabidopsis genome is differentially expressed during the early stage of infection by *Botrytis cinerea* (Windram et al., 2012). The dramatic transcription reprogramming is conferred by the concerted action of myriad transcription (co)factors (TFs) that function directly or indirectly to deploy their activity rapidly, transiently, spatially and hierarchically. In recent years, many TFs belonging to the AP2/ERF, NAC, MYB, WRKY, and bZIP (super)families have been identified to play important roles in regulating plant immune response against diverse pathogens (Eulgem and Somssich, 2007; Alves et al., 2013; Licausi et al., 2013; Nuruzzaman et al., 2013).

*Botrytis cinerea* is an airborne plant pathogen with a necrotrophic lifestyle attacking over 200 crop hosts worldwide (Williamson et al., 2007) and the interaction of tomato-*B. cinerea* has been developed as a useful pathosystem to study the molecular mechanism of plant immunity to necrotrophic fungal pathogens (Mengiste, 2012). Generally speaking, PTI but not ETI is effective in plant immunity to necrotrophic fungal pathogens such as *B. cinerea* (Mengiste, 2012). Several quantitative trait loci conferring resistance or susceptibility to *B. cinerea* have been identified and mapped in tomato (Finkers et al., 2007; Davis et al., 2009). Significant transcriptional reprogramming, metabolic and biochemical changes, and modification of signal pathways operated by different stress hormones such as ethylene (ET), salicylic acid (SA), jasmonic acid (JA), and abscisic acid (ABA) are involved in the response of tomato or its wild species to *B. cinerea* (Blanco-Ulate et al., 2013; Seifi et al., 2014; Smith et al., 2014; Camañes et al., 2015; Vega et al., 2015). During the infection process, *B. cinerea* can manipulate the antagonistic balance between the SA- and JA/ET-mediated signaling pathways and hijack the SA signaling pathway to accelerate disease development (El Oirdi et al., 2011; Rahman et al., 2012). Shortening in JA biosynthesis resulted in increased susceptibility to *B. cinerea* (Hind et al., 2011; Zhang S. et al., 2015), whereas ET-mediated signaling plays a positive role in immunity to *B. cinerea* (Francia et al., 2007; Lin et al., 2008; Nambeesan et al., 2012). It was shown that ABA regulates the immunity to *B. cinerea* in tomato through modulating the cuticle permeability and pectin composition in cell wall or suppressing the SA-mediated signaling pathway or the production of nitric oxide (Audenaert et al., 2002; Asselbergh et al., 2007; Curvers et al., 2010; Sivakumaran et al., 2016). A number of genes encoding receptor-like protein kinase TPK1b, transcriptional factors SHINE3, AIM1, SlDRW1, SlSRN1, SlSR1, and SlSR3L (Abuqamar et al., 2008, 2009; Buxdorf et al., 2014; Li et al., 2014a; Liu et al., 2014a,b), histone H2B monoubiquitination enzymes SlHUB1 and SlHUB2 (Zhang Y. et al., 2015), mitogen-activated protein kinase kinase SlMKK2 and SlMKK4 (Li et al., 2014b), phosphatidylinositol-phospholipase SlPLC2 (Gonorazky et al., 2016), NADPH oxidase SlRbohB (Li X. et al., 2015), 12-oxophytodienoate reductase SlOPR3 (Scalschi et al., 2015) and matrix metalloproteinase Sl3-MMP (Li D. et al., 2015) have been identified to play important roles in tomato immunity against *B. cinerea*. Enzymes involved in biosynthesis of vitamin B6 and trehalose-6-phosphate as well as concurrent over-activation of cytosolic glutamine synthetase and γ-aminobutyric acid shunt are also involved in tomato immune response to *B. cinerea* (Seifi et al., 2013; Zhang et al., 2014, 2016). Furthermore, simultaneous suppression of both polygalacturonase and expansin or accumulation of anthocyanin decreased the susceptibility of ripening fruits to *B. cinerea* (Cantu et al., 2008; Zhang et al., 2013). However, our knowledge on the molecular mechanism regulating the plant immunity to necrotrophic fungal pathogens is relatively lagging, as compared to the progress in plant immunity to (hemi)biotrophic pathogens.

The AP2/ERF superfamily is a large family with more than 100 members in plants [e.g., 147 in Arabidopsis (Nakano et al., 2006) and 139 in rice (Sharoni et al., 2011)] and represents a unique group of plant-specific TFs (Riechmann et al., 2000). A common structural feature of the proteins encoded by this superfamily is the presence of a highly conserved DNA-binding domain, called AP2 domain, containing 58 or 59 amino acids involved in the high-affinity binding to target DNA sequences (Ohme-Takagi and Shinshi, 1995). According to the numbers and characteristics of the AP2 domains, the AP2/ERF superfamily is basically divided into three families, e.g., AP2 family with two AP2 domains, ERF/DREB family with one AP2 domain and RAV family with a B3 DNA-binding domain (Riechmann et al., 2000; Nakano et al., 2014). The ERF/DREB family falls mainly into three subfamilies: ERFs (ethylene response factors), DREBs (dehydration-responsive element binding proteins) and the CBF (C-repeat binding factor) family (Nakano et al., 2014). Biochemical evidence indicates that the ERF proteins can specifically bind to a *cis*-element called GCC box (AGCCGCC) (Ohme-Takagi and Shinshi, 1995; Buttner and Singh, 1997), which is present in the promoter regions of many ethylene-regulated defense genes, and function as transcriptional activators or repressors (Fujimoto et al., 2000). It was also found that several members of the ERF family contain phosphorylation sites in C-terminals and need posttranslational modification for their biochemical activity and biological functions (Meng et al., 2013).

Extensive studies with loss-of-function and gain-of-function mutants in different plant species have demonstrated the importance of the ERF proteins during plant development and adaptation to abiotic stress conditions (Licausi et al., 2013). Meanwhile, a large number of functional investigations have also suggested that the ERF family play critical roles in plant response to biotic stresses (Licausi et al., 2013). Among the 12 major groups classified based on the type of AP2 domain (Nakano et al., 2006), the B3 group (also called as IX group) has been shown to play important roles in regulating defense responses in different plants against pathogens. For example, overexpression of Arabidopsis *ERF1* or *AtERF2*, tobacco *ERF5* or *OPBP1*, tomato *Pti4* or *Pti5*, rice *OsERF922* or *OsBIERF3*, wheat *TaPIEP1* and *Medicago truncatula MtERF1-1* in transgenic plants resulted in enhanced resistance against a variety of diseases caused by necrotrophic or biotriophic fungal and bacterial pathogens (Solano et al., 1998; He et al., 2001; Berrocal-Lobo et al., 2002; Gu et al., 2002; Fischer and Droge-Laser, 2004; Guo et al., 2004; Cao et al., 2006; Anderson et al., 2010; Dong et al., 2010; Son et al., 2011; Liu et al., 2012). The Arabidopsis ERF1, ERF5, ERF6, AtERF14, and ORA59 have been shown to act as regulators of the JA/ET signaling pathway that is required for resistance against necrotrophic fungal pathogens including *B. cinerea* (Lorenzo et al., 2003; Berrocal-Lobo and Molina, 2004; Oñate-Sánchez et al., 2007; Moffat et al., 2012). Together, at least 7 (ERF1, AtERF2, AtERF5, AtERF6, AtERF14, ORA59, and AtERF104) out of 17 members in the B3 group of the Arabidopsis ERF family have functions in regulating defense response against pathogen infections, demonstrating the importance of this group in plant disease resistance.

Recent genome-wide bioinformatics analyses identified a total of 146 members in the tomato AP2/ERF superfamily, of which 77 members belong to the ERF family (Sharma et al., 2010; Pirrello et al., 2012; Liu et al., 2016). Expression profiling analyses revealed that a large set of the tomato ERF genes are differentially induced by developmental cues, hormones and various stress factors (Gu et al., 2000; Tournier et al., 2003; Sharma et al., 2010; Pirrello et al., 2012). Functional studies have characterized a number of tomato ERF genes, e.g., *SlERF1, Sl-ERF2, TSRF1, SlAP2a, Sl-ERF.B3, SlERF6, SlERF36*, and *SlERF52*, that play important roles in regulating growth and developmental processes including seed germination, seedling development, stomatal density, fruit ripening, and flower pedicel abscission (Pirrello et al., 2006; Li et al., 2007; Zhang et al., 2008, 2009; Chung et al., 2010; Lee et al., 2012; Liu et al., 2013, 2016; Upadhyay et al., 2013; Liu M. et al., 2014; Nakano et al., 2014). Whereas *TERF1, TERF2, TSRF1, JERF1, JERF3, LeERF3b, Sl-ERF.B.3*, and *ERF5* were found to be involved in regulating abiotic stress response (Huang et al., 2004; Wang et al., 2004; Zhang et al., 2004a,b, 2007, 2010; Chen et al., 2008; Wu et al., 2008; Quan et al., 2010; Zhang and Huang, 2010; Tian et al., 2011; Pan et al., 2012; Klay et al., 2014), *Pti4, Pti5, Pti6, TSRF1*, and *SlERF3* have been demonstrated to play key roles in regulating defense responses against pathogens and insect pests (Zhou et al., 1997; He et al., 2001; Gu et al., 2002; Wu et al., 2002, 2015; Zhang et al., 2004b, 2007; Pan et al., 2010). Among the characterized tomato ERF genes, seven of them including *Pti4, Pti5, LeERF1, LeERF4* (*Sl-ERF.B3*), *TSRF1, TERF1*, and *ERF5* are members of the B3 group (Zhou et al., 1997; Tournier et al., 2003; Huang et al., 2004; Zhang et al., 2004a,b, 2007; Pan et al., 2012; Liu et al., 2013, 2016; Liu M. et al., 2014). Here, we performed virus-induced gene silencing (VIGS)-based functional analyses of 18 members in the B3 group members of the tomato ERF family to explore their possible involvement in disease resistance against *B. cinerea*. Our results data indicate that SlERF.A1, SlERF.A3, SlERF.B4, and SlERF.C3 positively regulate resistance of tomato plants against *B. cinerea*.

## Materials and Methods

### Plant Growth Condition and Treatments

Tomato (*Solanum lycopersicum*) cv. Suhong 2003 was used for all experiments. Tomato and *Nicotiana benthamiana* plants were grown in plastic pots containing compost soil mix (perlite: vermiculite: plant ash = 1: 6: 2) under 10 h fluorescent light (200 μE m^2^ s^-1^) at 22 ∼ 24°C with a 14 h light/10 h dark cycle. Generally, 2-week-old tomato seedlings were used for VIGS assays and 4-week-old plants were used for gene expression experiments. For analyses of gene expression in response to hormone treatments, tomato plants were treated by foliar spraying with 100 μM methyl jasmonate (MeJA), 1 mM SA, 100 μM 1-aminocyclopropane-1-carboxylic acid (ACC, a precursor of ethylene) in solutions containing 0.1% ethanol and 0.02% Tween-20. Plants treated with same volume of solution containing 0.1% ethanol and 0.02% Tween-20 were used as a control. For analyses of gene expression in response to *B. cinerea*, whole plant inoculation assays along with a corresponding mock-inoculation control were performed (see below for detail). Leaf samples were collected from hormone-treated or pathogen-inoculated plants at different time points as indicated after treatment or inoculation and stored at -80°C until use.

### Vector Construction and VIGS Assays

According to the predicted cDNA and available full-length cDNAs, VIGS fragments of 250–330 bp (Supplementary File 1) were designed at the 5′ ends of the selected tomato B3 group ERF genes and amplified with gene-specific primers with different restriction enzyme sites (Supplementary Table S1) from cDNAs synthesized from total RNA prepared from tomato leaf samples. After cloning and sequencing, the VIGS fragments were cloned into pTRV2 vector (Liu et al., 2002), yielding pTRV2-SlERF-A, B and Cs, which were then introduced into *Agrobacterium tumefaciens* strain GV3101 by electroporation using GENE PULSER II Electroporation System (Bio-Rad Laboratories, Hercules, CA, USA). Agrobacteria carrying pTRV2-GUS (as a negative control) or pTRV2-SlERF-A, B and Cs were cultivated in YEP medium (10 g/l peptone, 10 g/l yeast extract, 5 g NaCl/l, 50 μg/ml rifampicin, 50 μg/ml kanamycin, and 25 μg/ml gentamicin) for 36 h with continuous shaking in a 28°C incubator. Cells were collected by centrifugation and resuspended in infiltration buffer (10 mM MgCl_2_, 150 μM acetosyringone, MES, pH5.7). The agrobacteria carrying pTRV2-GUS or pTRV2-SlERF-A, B and Cs were mixed with the agrobacteria carrying pTRV1 in a ratio of 1:1 and maintained at OD_600_ = 1.5 for 3 h at room temperature. The mixed agrobacteria suspension was infiltrated into the abaxial surface of the 2-week-old seedlings using 1 mL needleless syringes. Leaf samples were collected at 2 weeks after VIGS infiltration and used for analysis of the silencing efficiency and specificity by qRT-PCR using gene-specific primers (Supplementary Table S1).

### Plant Inoculation and Disease Assays

*Botrytis cinerea* was cultivated on 2 × V8 agar (36% V8 juice, 0.2% CaCO_3_, 2% agar) at 22°C. Spores were collected from 10-day-old plates by rinsing the culture with 1% maltose buffer, filtered through bi-layered cheesecloth and adjusted to 2 × 10^5^ spores/ml for inoculation (Li et al., 2014a). For gene expression analyses, 4-week-old plants were inoculated by foliar spraying with spore suspension or with same volume of 1% maltose buffer as a mock-inoculation control and leaf samples were collected at different time points after inoculation. For disease assays, simplified detached leaf inoculation assay and whole plant inoculation assay were performed. In the detached leaf inoculation assays, leaves were detached from at least 10 VIGS-infiltrated plants at 4 weeks after VIGS infiltration and inoculated by dropping 5 μl of spore suspension on leaf surface. In the whole plant inoculation assays, 6-week-old plants were inoculated by foliar spraying with spore suspension. The inoculated leaves and plants were kept at 22°C in sealed containers to retain the moist conditions favorable for disease development. Lesion sizes on inoculated detached leaves were measured. Fungal growth *in planta* was analyzed by amplification of the transcripts of *B. cinerea* Actin gene as a marker using a pair of primers *BcActin*-F and *BcActin*-R (Supplementary Table S1) (Li et al., 2014b). Relative fungal growth was expressed as folds of the transcript levels of *BcActin* vs the transcript levels of a tomato *Actin* gene.

*Pseudomonas syringae* pv. *tomato* DC3000 was grown overnight in King’s B (KB) liquid medium containing rifampicin at 50 μg/ml. Cells were collected and resuspended in 10 mM MgCl_2_ to OD_600_ = 0.0002 for plant inoculation. Plants were vacuum infiltrated with suspension of *Pst* DC3000 or with MgCl_2_ solution as a mock inoculation control at 4 weeks after VIGS treatment. The inoculated plants were kept in a sealed container to maintain high humidity (RH > 90%) and disease progress was observed daily. For measurement of *in planta* bacterial growth, disks (6 mm in diameter) from leaves of *Pst* DC3000-inoculated plants were surface sterilized in 75% ethanol for 10 s, homogenized in 200 μl of 10 mM MgCl_2_, diluted in 10 mM MgCl_2_, and plated on KB agar plates containing 50 μg/ml rifampicin. The plates were incubated at 28°C and the bacterial colonies were counted 3 days after incubation.

### Subcellular Localization Assays

Coding sequences of *SlERF.A1, SlERF.A3, SlERF.B4*, and *SlERF.C3* were amplified using gene-specific primers SlERF.A1-1F/1R, SlERF.A3-1F/1R, SlERF.B4-1F/1R, or SlERF.C3-12F/1R (Supplementary Table S1), respectively. After cloning and confirmation by sequencing, the coding sequences were released from recombinant plasmids and inserted into pFGC-Egfp vector, yielding pFGC-Egfp:SlERF.A1, pFGC-Egfp:SlERF.A3, pFGC-Egfp:SlERF.B4, and pFGC-Egfp:SlERF.C3. These sequence-verified constructs and the empty vector pFGC-Egfp were introduced into *A. tumefaciens* strain GV3101. Four-week-old *N. benthamiana* plants were infiltrated with agrobacteria carrying different constructs and allowed to grow at 25°C for 48 h. Fluorescence was excited at 488 nm and detected using a 500–530 nm emission filter preformed with confocal laser scanning microscope (Model LSM 510 META, Zeiss, Oberkochen, Germany).

### Transactivation Activity Assays

The coding sequences of *SlERF.A1, SlERF.A3, SlERF.B4*, and *SlERF.C3* were amplified using gene-specific primers SlERF.A1-2F/2R, SlERF.A1-2F/2R, SlERF.B4-2F/2R, and SlERF.C3-2F/2R (Supplementary Table S1), respectively, and cloned into pBD-GAL4Cam vector, yielding pBD-SlERF.A1, pBD-SlERF.A3, pBD-SlERF.B4, and pBD-SlERF.C3. The recombinant plasmids and the pBD empty vector (a negative control) were transformed into yeast strain AH109. The transformed yeasts were cultivated on the SD/Trp^-^ and SD/Trp^-^His^-^ medium for 3 days at 28°C, followed by addition of x-α-gal. Transactivation activity of the fused proteins was evaluated according to the growth situation and production of blue pigments after the addition of x-α-gal of the transformed yeast cells on the SD/Trp^-^/His^-^ medium.

### Analyses of Gene Expression

Gene-specific primers were designed using Primer Premier 6 and located near the 3′ ends of the target genes (Supplementary Table S1). Total RNA was extracted using TRIzol reagent (Invitrogen, Shanghai, China) and treated with RNase-free DNase (TaKaRa, Dalian, China). First-strand cDNA was synthesized from 1 μg total RNA using AMV reverse transcriptase (TaKaRa, Dalian, China). Each qRT-PCR reaction contained 12.5 μl SYBR Premix Ex Taq (TaKaRa, Dalian, China), 0.1 μg cDNA and 7.5 pmol of each gene-specific primer in a final volume of 25 μl, and performed in a CFX96 real-time PCR detection system (Bio-Rad, Hercules, CA, USA). Three independent biological replicates were used for analyses and relative expression levels were calculated using the 2^-ΔΔCT^ method. A tomato actin gene was used as an internal control and relative expression levels of the target genes were shown as folds of the expression level of the actin gene.

### *In situ* Detection of H_2_O_2_

*In situ* detection of H_2_O_2_ in leaves of mock- and *B. cinerea*-inoculated plant was carried out by the 3, 3-diaminobenzidine (DAB) staining method (Thordal-Christensen et al., 1997). Accumulation of H_2_O_2_ in stained leaves was visualized by a digital camera.

### Statistical Analysis

Experiments were repeated three times and three replicates were included in each experiment. Data obtained from three independent experiments were subjected to statistical analysis using the Student’s *t*-test and he probability values of *p* < 0.05 were considered as significant difference.

## Results

### The B3 Group of the Tomato ERF Family and VIGS-based Silencing of the Selected B3 Group ERF Genes

A total of 28 members of the B3 group in tomato ERF family were previously identified (Sharma et al., 2010; Pirrello et al., 2012; Liu et al., 2016). However, locus Solyc09g089910 corresponding to *SlERF59* (Sharma et al., 2010) was not included as a B3 group member (Pirrello et al., 2012; Liu et al., 2016). Solyc09g089910 is phylogenetically related to subgroup IXc (Pirrello et al., 2012; Liu et al., 2016) and was named as SlERF.C11 (Liu et al., 2016). Thus, the B3 group of the tomato ERF family harbors 29 members in total, among which 5, 13, and 11 are assigned into IXa, IXb, and IXc subgroups, respectively.

**FIGURE 1:**
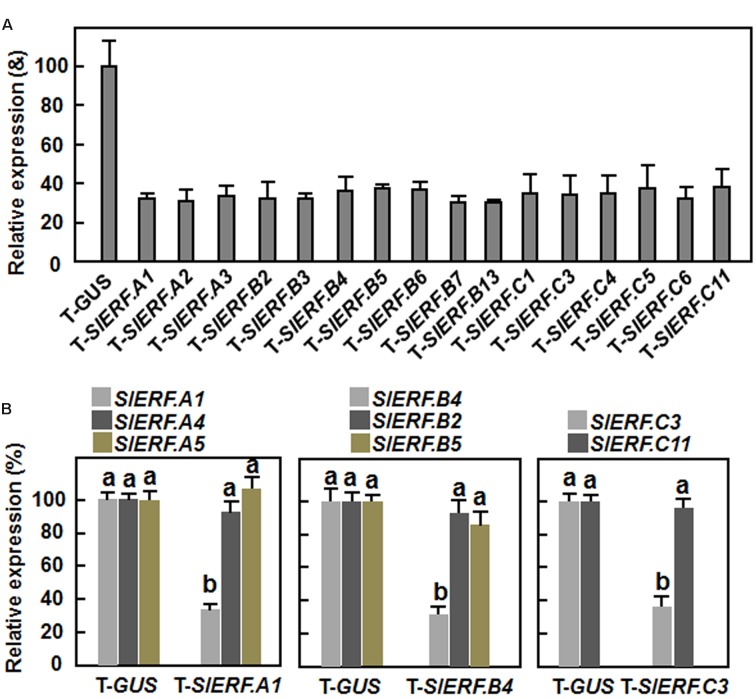
**Silencing efficiency and specificity for target genes.** Two-week-old seedlings were infiltrated with agrobacteria carrying different virus-induced gene silencing (VIGS) constructs and leaf samples were collected at 2 weeks after VIGS infiltration. **(A)** Silencing efficiency for target genes in VIGS-infiltrated plants. **(B)** Silencing specificity for target genes in VIGS-infiltrated plants. Transcript levels of the target genes were analyzed by qRT-PCR and data were normalized with a tomato actin gene. Silencing efficiency was calculated by comparing the transcript levels of each *SlERF* gene in corresponding pTRV2-SlERF-infiltrated plants to those in pTRV2-GUS-infiltrated plants. The transcript levels for each *SlERF* gene in the pTRV2-GUS-infiltrated plants were set as 100%. The transcript levels of each *SlERF* gene in VIGS-infiltrated plants were shown above the columns. Data are the mean ± SD from three independent experiments and different letters above the columns indicate significant difference at *p* < 0.05 level between pTRV2-SlERF- and pTRV2-GUS-infiltrated plants.

To explore the possible involvement of the B3 group ERF genes in tomato resistance against *B. cinerea*, 18 members, among which 16 members had available full-length cDNAs and two members did not have full-length cDNAs, were selected for study. Fragments of 250–330 bp in size at the 5′ ends of the genes for each of these selected B3 group ERF genes were amplified and constructed into pTRV2 vectors (Supplementary File 1). Standard VIGS assays were performed on 2-week-old tomato seedlings (Liu et al., 2002). In our VIGS assays, efficiency of the VIGS protocol was >85%, estimated by the appearance of bleaching phenotype, which was resulted from silencing of a phytoene desaturase (PDS) gene as a positive control marker (Liu et al., 2002), in pTRV2-PDS-infiltrated plants. Silencing efficiency for each of the target genes selected under our experimental conditions was ∼60% (**Figure 1A**), as estimated by qRT-PCR analysis of the transcript levels for the target genes in pTRV2-SlERFs-infiltrated plants and compared with that in the pTRV2-GUS-infiltrated negative control plants. As *SlERF.A1, SlERF.B4*, and *SlERF.C3* were later shown to be involved in resistance to *B. cinerea* (see below), silencing specificity of *SlERF.A1, SlERF.B4*, and *SlERF.C3* genes in pTRV2-SlERF.A1-, pTRV2-SlERF.B4-, or pTRV2-SlERF.C3-infiltrated plants was further evaluated. In a phylogenetic tree constructed with cDNA sequences of the B3 group ERF genes, *SlERF.A1* is closely related to *SlERF.A4* and *SlERF.A5, SlERF.B4* shows high levels of identity to *SlERF.B2* and *SlERF.B5*, and *SlERF.C3* is highly related to *SlERF.C11* (Supplementary Figure S1). As shown in **Figure 1B**, the transcript levels of *SlERF.A4* and *SlERF.A5* in pTRV2-SlERF.A1-infiltrated plants, *SlERF.B2* and *SlERF.B5* in pTRV2-SlERF.B4-infiltrated plants and *SlERF.C11* in pTRV2-SlERF.C3-infiltrated plants were comparable to those in pTRV2-GUS-infiltrated plants. These data indicate that silencing of *SlERF.A1, SlERF.B4*, or *SlERF.C3* did not significantly affect the expression of their closely related genes.

### Silencing of *SlERF.A3, SlERF.B1*, or *SlERF.C2* Affected Vegetative Growth

**FIGURE 2:**
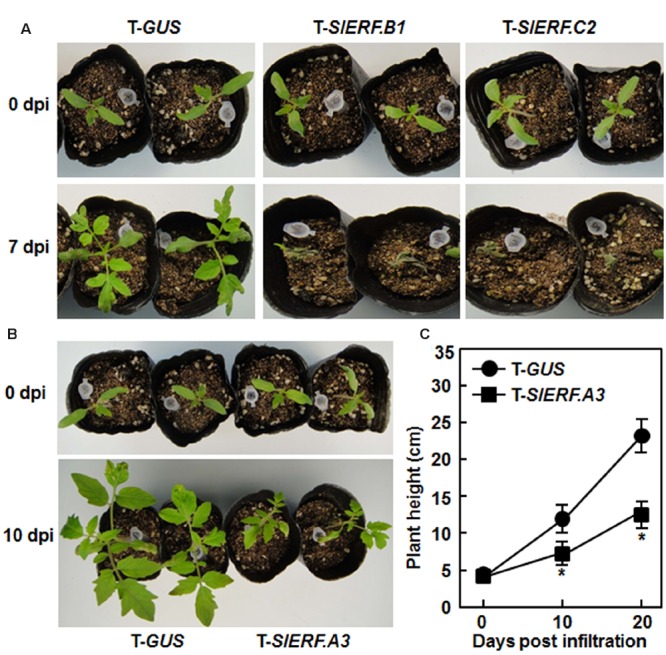
**Silencing of *SlERF.A3, SlERF.B1*, and *SlERF.C2* affected vegetative growth of VIGS-infiltrated plants. (A)** Silencing of *SlERF.B1* or *SlERF.C2* led to death of the pTRV2-SlERF.B1- and pTRV2-SlERF.C2-infiltrated plants. **(B)** and **(C)** Silencing of *SlERF.A3* suppressed vegetative growth of the pTRV2-SlERF.A3-infiltrated plants. Heights of the pTRV2-SlERF.A3- and pTRV2-GUS-infiltrated plants grown under same condition were measured at 10 and 20 days after VIGS infiltration. Data presented in **(C)** are the mean ± SD from three independent experiments. ^∗^Below the line in **(C)** indicates significant difference at *p* < 0.05 between the pTRV2-SlERF.A3- and pTRV2-GUS-infiltrated plants. dpi, days post-infiltration.

During our repeated experiments, the pTRV2-SlERF.B1- and pTRV2-SlERF.C2-infiltrated plants died within 7 days after VIGS infiltration while the pTRV2-GUS-infiltrated plants grew normally (**Figure 2A**), implying that silencing of either *SlERF.B1* or *SlERF.C2* had lethal effect on growth of tomato plants. Furthermore, silencing of *SlERF.A3* (*Pti4*) suppressed significantly the growth of pTRV2-SlERF.A3-infiltrated plants (**Figure 2B**), as the heights of the pTRV2-SlERF.A3-infiltrated plants were approximately 55% of the pRTV2-GUS-infiltrated plants at 10 and 20 days after VIGS infiltration (**Figure 2C**), indicating that silencing of *SlERF.A3* had an effect on the growth of tomato plants. During a period of 6 weeks, no significant defect in growth and development was observed for plants that were VIGS infiltrated and silenced for one of the other selected B3 group member ERF genes.

### Silencing of *SlERF.A1, SlERF.B4, SlERF.C3*, or *SlERF.A3* Led to Decreased Resistance to *B. cinerea*

**FIGURE 3:**
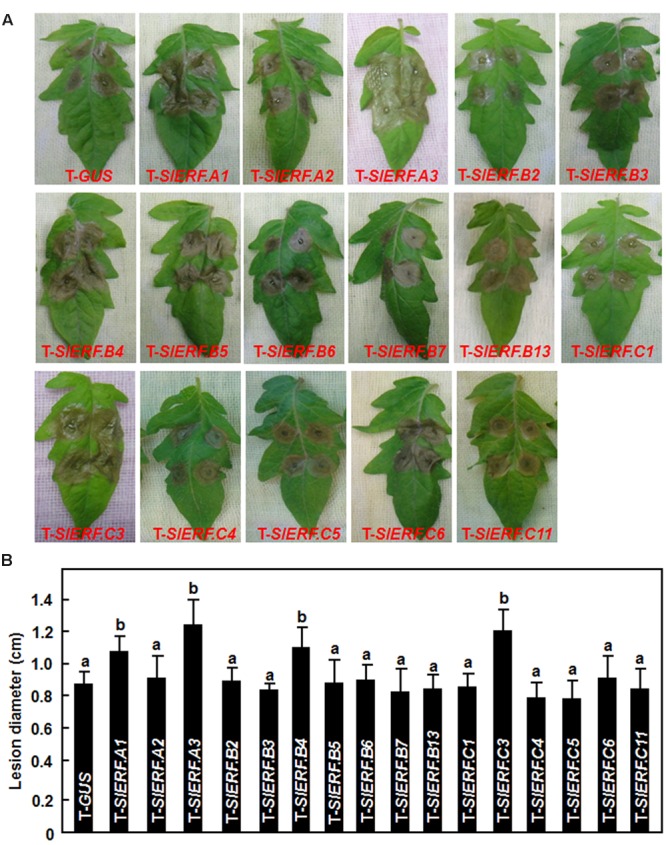
***Botrytis cinerea*-caused disease phenotype on leaves of VIGS-infiltrated plants with silencing of individual B3 group Ethylene-Responsive Factors (ERF) gene.** Two-week-old tomato seedlings were infiltrated with agrobacteria carrying pTRV2-GUS or pTRV2-SlERFs constructs and fully expanded leaves were detached from pTRV2-GUS- or pTRV2-SlERF-infiltrated plants at 4 weeks after VIGS infiltration for disease assays. Inoculation with *B. cinerea* was done by dropping 5 μl of spore suspension (2 × 10^5^ spores/ml). **(A)** Disease symptom on detached leaves at 3 days post inoculation (dpi). **(B)** Lesion sizes in leaves of the pTRV2-GUS- or pTRV2-SlERF-infiltrated plants at 3 days after inoculation. At least 10 leaves from ten individual plants were used for each experiment. Data presented **(B)** are the means ± SD from three independent experiments and different letters above the columns indicate significant differences at *p* < 0.05 level.

**FIGURE 4:**
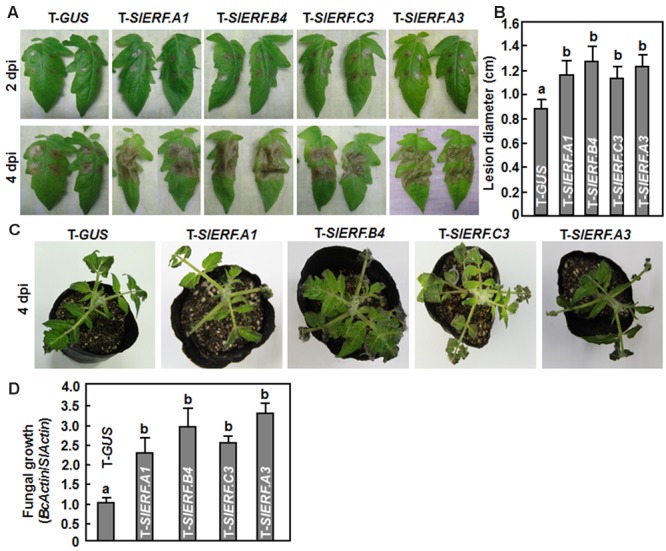
**Silencing of *SlERF.A1, SlERF.A3, SlERF.B4*, and *SlERF.C3* resulted in increased susceptibility to *B. cinerea*.** Two-week-old seedlings were infiltrated with agrobacteria carrying pTRV2-SlERF.A1, pTRV2-SlERF.A3, pTRV2-SlERF.B4, pTRV2-SlERF.C3, or pTRV2-GUS and disease assays were performed on detached leaves and whole plants at 4 weeks after VIGS infiltration. Inoculation with *B. cinerea* was done by dropping 5 μl of spore suspension (2 × 10^5^ spores/ml) on surface of the detached leaves or foliar spraying onto leaves of whole plants. **(A)** Disease symptom and progress on detached leaves of pTRV2-SlERF.A1-, pTRV2-SlERF.A3, pTRV2-SlERF.B4-, pTRV2-SlERF.C3-, or pTRV2-GUS-infiltrated plants at 2 and 4 days post inoculation (dpi). **(B)** Lesion sizes in leaves of the pTRV2-SlERF.A1-, pTRV2-SlERF.A3, pTRV2-SlERF.B4-, pTRV2-SlERF.C3-, or pTRV2-GUS-infiltrated plants at 4 days post inoculation. At least 10 leaves from ten individual plants were used for each experiment. **(C)** Disease phenotype on representative inoculated plants at 4 days post inoculation. **(D)** Growth of *B. cinerea* in inoculated leaves of pTRV2-SlERF.A1-, pTRV2-SlERF.A3, pTRV2-SlERF.B4-, pTRV2-SlERF.C3-, or pTRV2-GUS-infiltrated plants. Fungal growth *in planta* was assumed at 4 days after inoculation by qRT-PCR analyzing the transcript levels of *B. cinerea BcActinA* gene using *SlActin* gene. Relative fungal growth was shown as folds of transcript levels of *BcActin* compared to *SlActin*. Data presented in **(B)** and **(C)** are the means ± SD from three independent experiments and different letters above the columns indicate significant differences at *p* < 0.05 level.

Because of the lethality of the pTRV2-SlERF.B1- or pTRV2-SlERF.C2-infiltrated plants, a total of 16 B3 group ERF genes were further examined using VIGS-based phenotyping for their involvement in disease resistance against *B. cinerea*. In our experiments, *B. cinerea*-caused lesions on leaves from pTRV2-SlERF.A2-, pTRV2-SlERF.B2-, pTRV2-SlERF. B3-, pTRV2-SlERF.B5-, pTRV2-SlERF.B6-, pTRV2-SlERF.B7-, pTRV2-SlERF.B13-, pTRV2-SlERF.C1-, pTRV2-SlERF.C4-, pTRV2-SlERF.C5-, pTRV2-SlERF.C6-, and pTRV2-SlERF. C11-infiltrated plants were similar to that on leaves from pTRV2-GUS-infiltrated plants (**Figure 3**), implying that these B3 group ERF genes may not be involved in disease resistance against *B. cinerea*. However, *B. cinerea*-caused lesions caused on leaves from pTRV2-SlERF.A1-, pTRV2-SlERF.A3-, pTRV2-SlERF.B4-, and pTRV2-SlERF.C3-infiltrated plants were significantly larger than the lesions on leaves from the pTRV2-GUS-infiltrated plants (**Figure 3A**) at 3 days after inoculation (**Figure 3B**), indicating that *SlERF.A1, SlERF.A3, SlERF.B4*, and *SlERF.C3* play roles in disease resistance against *B. cinerea*. Because the functions of *SlERF.A1, SlERF.B4*, and *SlERF.C3* have not been characterized previously, we further analyzed and compared the *B. cinerea*-provoked disease progress and *in planta* fungal growth between the pTRV2-SlERF.A1-, pTRV2-SlERF.B4-, and pTRV2-SlERF.C3-infiltrated plants and the pTRV2-GUS-infiltrated plants. As shown in **Figure 4A**, separate *B. cinerea*-caused lesions were observed; however, sizes of lesions on leaves from pTRV2-SlERF.A1-, pTRV2-SlERF.A3-, pTRV2-SlERF.B4-, and pTRV2-SlERF.C3-infiltrated plants were larger than that on leaves from pTRV2-GUS-infiltrated plants at 2 day after inoculation. At 4 days after inoculation, the lesions merged into large necrotic area on leaves from pTRV2-SlERF.A1-, pTRV2-SlERF.A3-, pTRV2-SlERF.B4-, and pTRV2-SlERF.C3-infiltrated plants, whereas lesions on leaves from pTRV2-GUS-infiltrated plants remained separated (**Figure 4A**). The lesions on leaves from pTRV2-SlERF.A1-, pTRV2-SlERF.A3-, pTRV2-SlERF.B4-, and pTRV2-SlERF.C3-infiltrated plants showed 32, 39, 44, and 30% of increase over that in pTRV2-GUS-infiltrated plants (**Figure 4B**). In whole plant disease assays, the pTRV2-SlERF.A1-, pTRV2-SlERF.B4-, pTRV2-SlERF.C3-, and pTRV2-SlERF.A3-infiltrated plants showed severer disease than the pTRV2-GUS-infiltrated plants (**Figure 4C**). qRT-PCR analysis of the transcript levels of the *B. cinerea* actin gene *BcActin*, an indication of the rate of fungal growth *in planta*, in leaves of the inoculated whole plants, showed that the fungal growth in the pTRV2-SlERF.A1-, pTRV2-SlERF.B4-, pTRV2-SlERF.C3-, and pTRV2-SlERF.A3-infilrated plants were significantly increased, resulting in increases of 1.3, 1.9, 1.5, and 2.2-folds at 4 days after inoculation over that in the pTRV2-GUS-infiltrated plants (**Figure 4D**). These data demonstrate that silencing of *SlERF.A1, SlERF.B4, SlERF.C3*, or *SlERF.A3* resulted in increased susceptibility to *B. cinerea* and thus these three B3 group ERF genes may function as positive regulators of disease resistance against *B. cinerea*.

### Silencing of *SlERF.A3* But Not *SlERF.A1, SlERF.B4*, or *SlERF.C3* Decreased the Resistance to *Pst* DC3000

**FIGURE 5:**
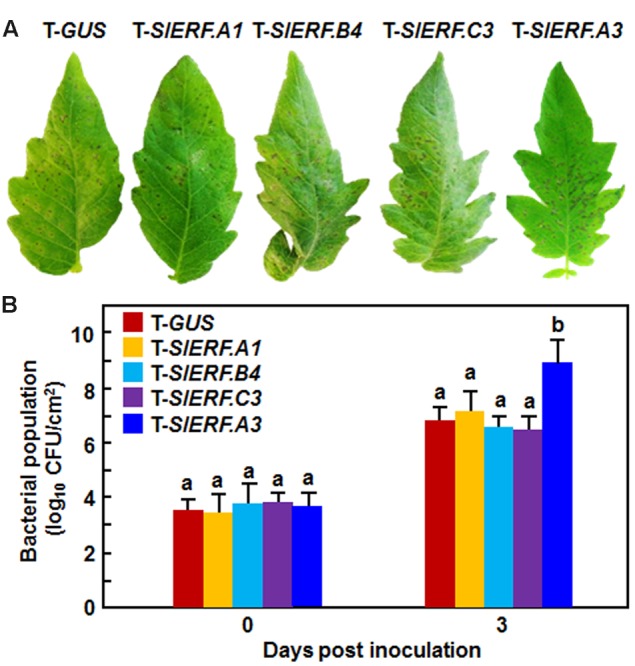
**Silencing of *SlERF.A3* but not *SlERF.A1, SlERF.B4*, and *SlERF.C3* decreased the resistance against *Pst* DC3000.** pTRV2-SlERF.A1-, pTRV2-SlERF.A3-, pTRV2-SlERF.B4-, pTRV2-SlERF.C3-, or pTRV2-GUS-infiltrated plants were inoculated with *Pst* DC3000 at 4 weeks after VIGS infiltration. Disease phenotype **(A)** and bacterial population **(B)** on leaves of SlERF.A1-, SlERF.A3-, SlERF.B4-, SlERF.C3-, and GUS-silenced plants were recorded and measured. Data presented **(B)** are the means ± SD from three independent experiments and different letters above the columns indicate significant differences at *p* < 0.05 level.

We further examined whether *SlERF.A1, SlERF.A3, SlERF.B4*, and *SlERF.C3* were also involved in resistance against *Pst* DC3000. As shown in **Figure 5A**, the *Pst* DC3000-caused disease symptom on and the *in planta* bacterial growth in the inoculated leaves of the pTRV2-SlERF.A1-, pTRV2-SlERF.B4-, and pTRV2-SlERF.C3-infilrated plants were similar to those in leaves of the pTRV2-GUS-infiltrated plants at 4 days after inoculation (**Figure 5B**). However, disease symptom on the inoculated leaves of the pTRV2-SlERF.A3-infiltrated plants was severer and the bacterial growth in leaves of the pTRV2-SlERF.A3-infiltrated plants showed ∼100 times higher, as compared to those in leaves of the pTRV2-GUS-infiltrated plants (**Figure 5**). These results indicate that *SlERF.A1, SlERF.B4*, or *SlERF.C3* may not have a function in tomato resistance against *Pst* DC3000 and that *SlERF.A3* is required for the resistance to *Pst* DC3000.

### Silencing of *SlERF.A1, SlERF.B4, SlERF.C3*, or *SlERF.A3* Attenuated Defense Response upon *B. cinerea* Infection

**FIGURE 6:**
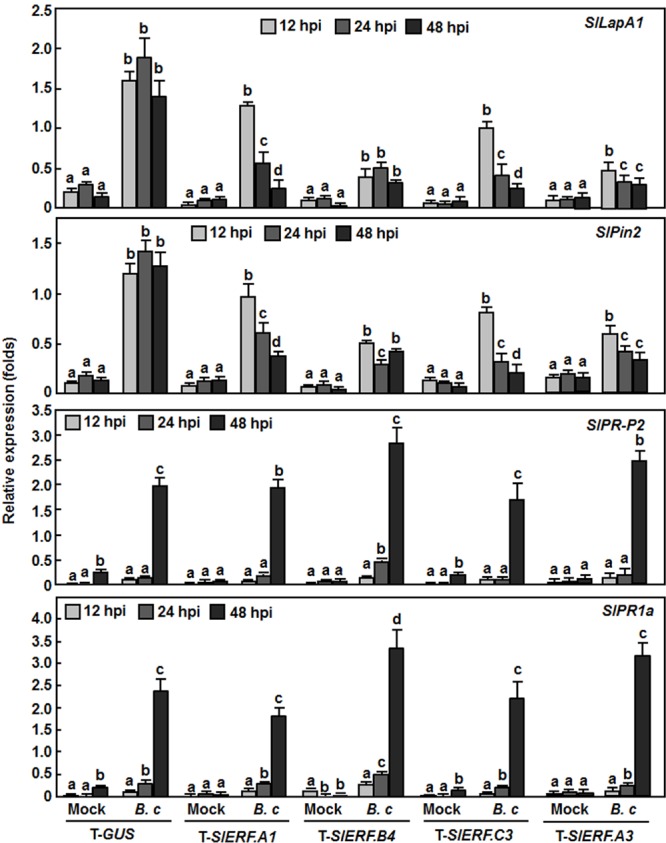
**Silencing of *SlERF.A1, SlERF.A3, SlERF.B4*, and *SlERF.C3* attenuated the *B. cinerea*-induced expression of JA/ET signaling pathway-regulated defense genes.** Two-week-old seedlings were infiltrated with agrobacteria carrying pTRV2-SlERF.A1, pTRV2-SlERF.A3, pTRV2-SlERF.B4, pTRV2-SlERF.C3, or pTRV2-GUS and inoculated by spraying with spore suspension of *B. cinerea* or with 1% maltose buffer as a mock control at 4 weeks after VIGS infiltration. Leaf samples were collected at 12, 24, and 48 h post inoculation (hpi) for analyzing expression of selected defense-related genes by qRT-PCR. Relative expression folds were calculated after normalization with actin transcript values. Data presented are the means ± SD from three independent experiments and different letters above the columns indicate significant difference at *p* < 0.05 level between pTRV2-SlERF- and pTRV2-GUS-infiltrated plants.

To explore whether silencing of *SlERF.A1, SlERF.B4*, and *SlERF.C3* affected the defense response, we analyzed and compared the expression levels of some well-known defense-related genes between the pTRV2-SlERF.A1-, pTRV2-SlERF.B4-, and pTRV2-SlERF.C3-infilrated plants and pTRV-GUS-infiltrated plants with or without inoculation with *B. cinerea*. *SlLapA1* and *SlPin2* were considered to be regulated by the JA/ET signaling pathway while *SlPR1a* and *SlPR-P2* were thought to be regulated by the SA-mediated signaling pathway (Kawazu et al., 2012). In mock-inoculation controls, the expression levels of these four selected defense-related genes were comparable between the pTRV2-SlERF.A1-, pTRV2-SlERF.B4-, pTRV2-SlERF.C3-, and pTRV2-SlERF.A3-infilrated plants and pTRV-GUS-infiltrated plants during a period of 48 h after mock-inoculation (**Figure 6**). However, the expression levels of *SlLapA1* and *SlPin2* in the pTRV2-SlERF.A1-, pTRV2-SlERF.B4-, pTRV2-SlERF.C3-, and pTRV2-SlERF.A3-infilrated plants were markedly reduced, leading to three–sixfolds of reduction as compared with those in the pTRV2-GUS-infiltrated plants at 12, 24, and 48 h after infection with *B. cinerea* (**Figure 6**, *upper two rows*). By contrast, the expression levels of *SlPR1a* and *SlPR-P2* in the pTRV2-SlERF.A1-, pTRV2-SlERF.B4-, pTRV2-SlERF.C3-, and pTRV2-SlERF.A3-infilrated plants at 12, 24, and 48 h after inoculation were markedly induced by *B. cinerea* but showed comparable induction patterns to those in the pTRV2-GUS-infiltrated plants (**Figure 6**, *lower two rows*). These results suggest that silencing of *SlERF.A1, SlERF.B4, SlERF.C3*, or *SlERF.A3* attenuated the *B. cinerea*-induced expression of the JA/ET signaling pathway-regulated defense-related genes but not affect the expression of the SA signaling pathway-regulated defense genes in tomato.

### Silencing of *SlERF.A1, SlERF.A3, SlERF.B4* ,or *SlERF.C3* Promoted H_2_O_2_ Accumulation upon *B. cinerea* Infection

**FIGURE 7:**
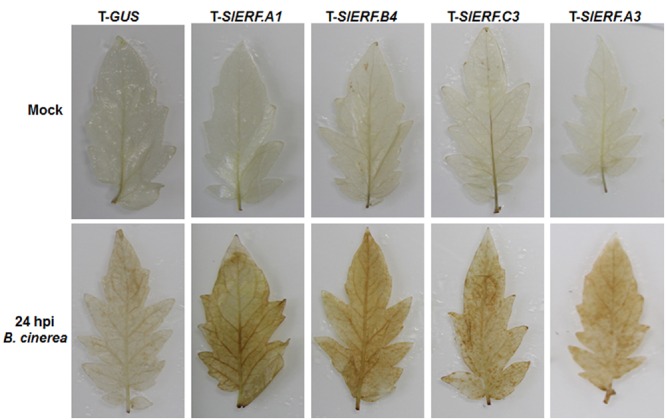
**Silencing of *SlERF.A1, SlERF.A3, SlERF.B4*, and *SlERF.C3* promoted the *B. cinerea*-induced H_2_O_2_ accumulation.** Two-week-old seedlings were infiltrated with agrobacteria carrying pTRV2-SlERF.A1, pTRV2-SlERF.A3, pTRV2-SlERF.B4, pTRV2-SlERF.C3, or pTRV2-GUS and inoculated by spraying with spore suspension of *B. cinerea* or with 1% maltose buffer as a mock control at 4 weeks after VIGS infiltration. Leaf samples were collected at 24 hpi and accumulation of H_2_O_2_ was detected by DAB staining. Two independent experiments were performed with similar results.

It is generally accepted that pathogen-induced accumulation of reactive oxygen species (ROS) may benefit the infection of necrotrophic fungal pathogens including by *B. cinerea* (Mengiste, 2012). To examine whether silencing of *SlERF.A1, SlERF.A3, SlERF.B4*, and *SlERF.C3* affected the accumulation of ROS, we analyzed and compared the accumulation of H_2_O_2_ between the pTRV2-SlERF.A1-, pTRV2-SlERF.A3-, pTRV2-SlERF.B4-, and pTRV2-SlERF.C3-infilrated plants and pTRV2-GUS-infiltrated plants with or without inoculation with *B. cinerea*. In mock-inoculated leaves, no significant accumulation of H_2_O_2_ was observed and no difference in H_2_O_2_ accumulation was seen between the pTRV2-SlERF.A1-, pTRV2-SlERF.A3-, pTRV2-SlERF.B4-, and pTRV2-SlERF.C3-infilrated plants and pTRV2-GUS-infiltrated plants (**Figure 7**). At 24 h after infection by *B. cinerea*, significant accumulation of H_2_O_2_ in inoculated leaves was detected in *B. cinerea*-infected leaves (**Figure 7**); however, more staining for H_2_O_2_ in *B. cinerea*-infected leaves of the pTRV2-SlERF.A1-, pTRV2-SlERF.A3-, pTRV2-SlERF.B4-, and pTRV2-SlERF.C3-infilrated plants was observed, as compared to that in *B. cinerea*-infected leaves of the pTRV2-GUS-infiltrated plants (**Figure 7**). These results indicate that silencing of *SlERF.A1, SlERF.A3, SlERF.B4*, or *SlERF.C3* promoted the accumulation of H_2_O_2_ upon infection of *B. cinerea*.

### Expression of *SlERF.A1, SlERF.B4, SlERF.C3*, and *SlERF.A3* Was Induced by *B. cinerea* and Defense Signaling Hormones JA and ACC

**FIGURE 8:**
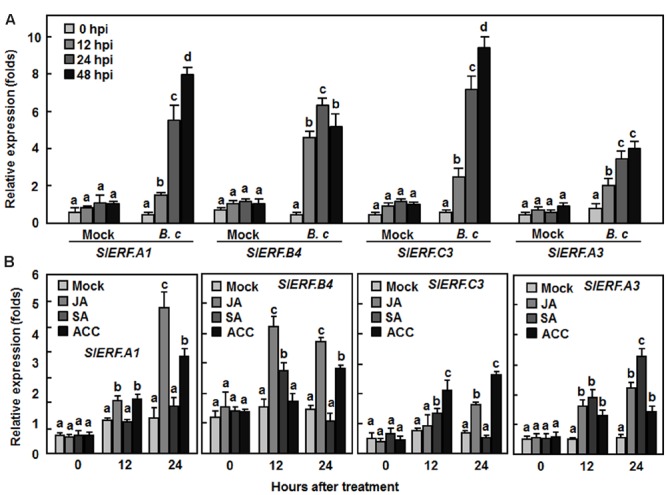
**Expression of *SlERF.A1, SlERF.A3, SlERF.B4*, and *SlERF.C3* induced by *B. cinerea* and by JA and ACC. (A)** Expression of *SlERF.A1, SlERF.A3, SlERF.B4*, and *SlERF.C3* in plants after inoculation with *B. cinerea*. Four-week-old plants were inoculated by foliar spraying with spore suspension of *B. cinerea* (*B. c*) or with 1% maltose buffer as a mock-inoculation control. **(B)** Expression of *SlERF.A1, SlERF.A3, SlERF.B4*, and *SlERF.C3* in plants after treatment with MeJA, SA, and ACC. Four-week-old plants were treated by foliar spraying with 100 μM MeJA, 1 mM SA, 100 μM ACC solutions or sterilized distill water as a control. Leaf samples were collected at indicated time points after inoculation or treatment for analysis of gene expression by qRT-PCR. Relative expression folds were calculated after normalization with actin transcript values. Data presented are the means ± SD from three independent experiments and different letters above the columns indicate significant difference at *p* < 0.05 level between pTRV2-SlERF- and pTRV2-GUS-infiltrated plants.

To gain further insights into the involvement of *SlERF.A1, SlERF.B4, SlERF.C3*, and *SlERF.A3* in disease resistance against *B. cinerea*, we analyzed their expression patterns in tomato plants after infection with *B. cinerea* or treatment with defense signaling hormones such as JA, SA, and ET-releasing precursor ACC. In mock-inoculated plants, the expression levels of *SlERF.A1, SlERF.B4, SlERF.C3*, and *SlERF.A3* were not changed markedly during a 48 hr experimental period (**Figure 8A**). However, infection of *B. cinerea* significantly induced the expression of the *SlERF.A1, SlERF.B4, SlERF.C3, and SlERF.A3* genes with similar patterns (**Figure 8A**). The expression levels in *B. cinerea*-inoculated plants showed 2.2–4.5, 4.7–6.7, 8.1–10.4, and 1.8–3.1-folds for *SlERF.A1, SlERF.B4, SlERF.C3*, and *SlERF.A3*, respectively, at 12, 24, and 48 h after inoculation, as compared with the levels in mock-inoculated plants (**Figure 8A**). Treatments with JA and ACC also induced the expression of *SlERF.A1, SlERF.B4, SlERF.C3, and SlERF.A3*, showing two–fourfolds of increases in JA- and ACC- treated plants, as compared with those in untreated control plants (**Figure 8B**). However, SA treatment did not affect the expression of *SlERF.A1* but induced significantly the expression of *SlERF.B4, SlERF.C3*, and *SlERF.A3* at 12 and/or 24 h after treatment (**Figure 8B**). These results indicate that expression of *SlERF.A1, SlERF.B4, SlERF.C3*, and *SlERF.A3* can be induced by infection of *B. cinerea* and treatment with JA, SA, and ACC.

### SlERF.A1, SlERF.A3, SlERF.B4, and SlERF.C3 Were Localized in Nucleus and Had Transactivation Activity in Yeast

**FIGURE 9:**
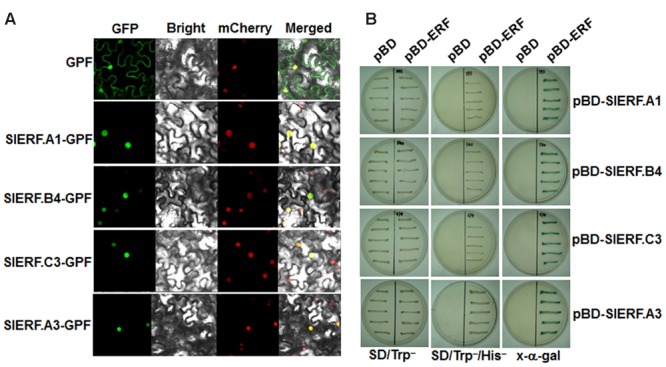
**Subcellular localization and transactivation activity of SlERF.A1, SlERF.A3, SlERF.B4, and SlERF.C3 proteins. (A)** Subcellular localization of SlERF.A1, SlERF.A3, SlERF.B4, and SlERF.C3 proteins. Agrobacteria carrying pFGC-Egfp-SlERF.A1, pFGC-Egfp-SlERF.A3, pFGC-Egfp-SlERF.B4, pFGC-Egfp-SlERF.C3, or pFGC-Egfp were infiltrated into leaves of *N. benthamiana* plants expressing a red nucleus marker RFP-H2B protein and the images were taken at 36 h after infiltration under dark field for green fluorescence (*left*) and red fluorescence (*middle right*), white field for cell morphology (*middle left*) and in combination (*right*), respectively. **(B)** Transactivation activity of SlERF.A1, SlERF.B4, and SlERF.C3 proteins in yeast. Yeast cells transformed with pBD-SlERF.A1, pBD-SlERF.A3, pBD-SlERF.B4, pBD-SlERF.C3 or pBD empty vector (as a negative control) were streaked on SD/Trp^-^ plates (*left*) or SD/Trp^-^His^-^ plates (*middle*) for 3 days at 28°C. The x-α-gal was added to the SD/Trp^-^His^-^ plates and kept at 28°C for 6 hr (*right*).

To examine the subcellular localization of SlERF.A1, SlERF.A3, SlERF.B4, and SlERF.C3 proteins, agrobacteria carrying pFGC-Egfp:SlERF.A1, pFGC-Egfp:SlERF.A3, pFGC-Egfp:SlERF.B4, pFGC-Egfp:SlERF.C3, and pFGC-Egfp (as a negative control) were infiltrated into leaves of 4-week-old *N. benthamiana* plants that expressed a red nuclear marker RFP–H2B protein (Chakrabarty et al., 2007). Confocal micrographs showed that SlERF.A1-GFP, SlERF.A3-GFP, SlERF.B4-GFP, and SlERF.C3-GFP were solely and clearly localized to the nucleus, co-localized with the known nucleus marker RFP–H2B protein (**Figure 9A**), whereas the GFP alone was detected in both the nucleus and cytoplasm (**Figure 9A**). These results demonstrate that the SlERF.A1, SlERF.A3, SlERF.B4, and SlERF.C3 proteins are localized to nucleus of the cells. Meanwhile, the transactivation activity of the SlERF.A1, SlERF.A3, SlERF.B4, and SlERF.C3 proteins were also determined using a yeast assay system. Yeast cells transformed with pBD-SlERF.A1, pBD-SlERF.A3, pBD-SlERF.B4, pBD-SlERF.C3 or pBD empty vector (as a negative control) grew well on SD/Trp^-^ medium (**Figure 9B**). However, only the pBD-SlERF.A1-, pBD-SlERF.A3-, pBD-SlERF.B4-, or pBD-SlERF.C3-transformed yeast cells grew well on SD/His^-^/Trp^-^ medium and produced blue pigments after addition of x-a-gal, whereas the pBD empty vector-transformed cells was unable to grow on SD/His^-^/Trp^-^ medium (**Figure 9B**). These results suggest that SlERF.A1, SlERF.A3, SlERF.B4, and SlERF.C3 proteins have transactivation activity in yeasts.

## Discussion

In the present study, VIGS-based functional analyses of 18 members in the B3 group of the tomato ERF family revealed that at least four members are required for disease resistance against *B. cinerea* and three members may have functions in regulating vegetative growth in tomato. Most importantly, our study presented evidence that SlERF.A1, SlERF.B4, and SlERF.C3, three previously uncharacterized members in B3 group of the ERF family, and SlERF.A3, a previously identified ERF with function in immunity to *Pst* DC3000 (Gu et al., 2002), positively regulate defense response against *B. cinerea*.

In Arabidopsis, several members in B3 group of the ERF family have been shown to function in defense response against *B. cinerea*, including ERF1, functionally redundant ERF5 and ERF6, and ORA59 (Berrocal-Lobo et al., 2002; Pré et al., 2008; Moffat et al., 2012). VIGS-based functional analyses of 16 members in B3 group of the tomato ERF family identified at least four members including *SlERF.A1, SlERF.A3* (*Pti4*), *SlERF.B4*, and *SlERF.C3* that are required for disease resistance against *B. cinerea* (**Figures 3** and **4**). The function of *SlERF.A3* (*Pti4*) was previously reported to be associated with the resistance against *Pst* DC3000 in overexpression transgenic Arabidopsis plants (Gu et al., 2002) and regulate defense response through interaction with Pto and binding to GCC and non-GCC boxes in promoters of defense genes (Zhou et al., 1997; Wu et al., 2002; Chakravarthy et al., 2003). Our VIGS experiments further support that *SlERF.A3* is required for the resistance of tomato plants against *Pst* DC3000 (**Figure 5**). However, the functions of the remaining three members *SlERF.A1, SlERF.B4*, and *SlERF.C3* are previously uncharacterized and we demonstrated that these three members are required for disease resistance against *B. cinerea*. This hypothesis is supported by several lines of evidence. Firstly, silencing of *SlERF.A1, SlERF.A3, SlERF.B4*, or *SlERF.C3* led to increased susceptibility to infection of *B. cinerea*, as evaluated by enhanced disease severity and *in planta* fungal growth in *SlERF.A1-, SlERF.A3*-, *SlERF.B4-*, and *SlERF.C3*-silenced plants (**Figures 3** and **4**). This is similar to the observation that the ORA59-silenced Arabidopsis plants showed increased susceptibility to *B. cinerea* (Pré et al., 2008). Secondly, expression of *SlERF.A1, SlERF.A3, SlERF.B4* and *SlERF.C3* was induced by infection of *B. cinerea* (**Figure 8A**), indicating that they are responsive to *B. cinerea*. Furthermore, the expression of *SlERF.A1, SlERF.A3, SlERF.B4*, and *SlERF.C3* was induced by MeJA, SA, and ACC (**Figure 8B**). Previously, the expression of *SlERF.A3* was shown to be induced by *Pst* DC3000 as well as by SA and ET (Thara et al., 1999; Gu et al., 2000). Recently, *SlERF.B6, SlERF.B7, SlERF.B8, SlERF.B9, SlERF.B10*, and *SlERF.B11* were identified as jasmonate-responsive ERFs, which are involved in biosynthesis of steroidal glycoalkaloids (Thagun et al., 2016). Thirdly, the *B. cinerea*-induced expression of the JA/ET signaling pathway-regulated defense genes *SlLapA1* and *SlPin2* in *SlERF.A1-, SlERF.A3*-, *SlERF.B4-*, and *SlERF.C3*-silenced plants was attenuated significantly (**Figure 6**). These data indicate that SlERF.A1, SlERF.A3, SlERF.B4, and SlERF.C3 might function in disease resistance against *B. cinerea* through the JA/ET signaling pathway, which is considered to mediate defense response against necrotrophic fungi including *B. cinerea* (Grant and Jones, 2009; Mengiste, 2012). In Arabidopsis, ERF1, ERF5, ERF6, AtERF14, and ORA59 were shown to act as regulators of the JA/ET signaling pathway (Lorenzo et al., 2003; Berrocal-Lobo and Molina, 2004; Oñate-Sánchez et al., 2007; Pré et al., 2008; Zarei et al., 2011; Moffat et al., 2012). Notably, the *B. cinerea*-induced expression of *SlLapA1* and *SlPin2* in *SlERF.A1*-, *SlERF.A3*-, and *SlERF.C3*-silenced plants decreased gradually with the progress of disease development, whereas the expression of these two genes in *SlERF.B4*-silenced plants maintained at relatively low level without alteration over the time (**Figure 6**). This may imply that distinct mechanisms modulated by SlERF.A1, SlERF.A3, SlERF.B4, and SlERF.C3 are involved in regulating defense response against *B. cinerea* through the JA/ET signaling. Lastly, it is well known that pathogen-induced ROS play different roles in immune response against pathogens with different infection styles. Generally, pathogen-induced ROS plays a signaling role in immunity against *Pst* DC3000 while this pathogen-induced ROS may benefit the infection by necrotrophic fungi such as *B. cinerea* (Mengiste, 2012). The significant accumulation of H_2_O_2_ in leaves of the *SlERF.A1*-, *SlERF.A3*-, *SlERF.B4*-, and *SlERF.C3*-silenced plants after infection of *B. cinerea* (**Figure 7**) may suggest that the function of SlERF.A1, SlERF.A3, SlERF.B4, and SlERF.C3 in defense response against *B. cinerea* links to ROS generation. Similar correlation of enhanced ROS accumulation and increased *B. cinerea* susceptibility were also observed in *SlSRN1*-, *SlMKK2*-, or *SlMKK4*-silenced tomato plants (Li et al., 2014b; Liu et al., 2014b).

However, the expression of two SA signaling pathway-regulated defense genes *SlPR1a* and *SlPR-P2* was not significantly suppressed in *SlERF.A1-, SlERF.B4-*, and *SlERF.C3*-silenced plants, as compared with their expressions in the control plants, after infection of *B. cinerea* (**Figure 6**), indicating that silencing of *SlERF.A1, SlERF.B4*, or *SlERF.C3* may not affect the SA signaling pathway. This can be partially corroborated by the observation that silencing of *SlERF.A1, SlERF.B4*, or *SlERF.C3* did not affect the phenotype of disease caused by *Pst* DC3000 (**Figure 5**). However, the involvement of SlERF.A1, SlERF.B4, and SlERF.C3 in SA signaling pathways cannot be excluded, as several members in B3 group of the Arabidopsis ERF family play roles in the modulating the balance between the JA/ET and SA signaling pathways to allow plants to mount an appropriate defense response against the attacking pathogen (Berrocal-Lobo et al., 2002; Moffat et al., 2012).

It is well known that some members of the AP2/ERF superfamily play important roles in regulating plant growth and development. However, evidence supporting the direct involvement of members in B3 group of the plant ERF family is limited. Recently, *SlERF.A2* (*LeERF1*) and *SlERF.B3* (*Sl-ERF.B.3*) were found to be involved in development and fruit ripening and softening in tomato (Li et al., 2007; Liu et al., 2013, 2016; Liu M. et al., 2014). In the present study, we found that silencing of *SlERF.B1* or *SlERF.C2* resulted in death of the pTRV2-SlERF.B1- and pTRV2-SlERF.C2-infiltrated plants within 7 days after VIGS infiltration (**Figure 2A**), even relative low concentrations of agrobacteria or relatively older plants were used for VIGS assays. Thus, it seems likely that both of SlERF.B1 and SlERF.C2 play roles in regulating vegetative growth of the tomato plants. On the other hand, it was previously reported that overexpression of *Pti4* led to inhibition of growth in transgenic Arabidopsis, displaying dwarf phenotype (Zhou et al., 1997). The growth inhibition in *Pti4*-overexpressing Arabidopsis plants might be due to the constitutively upregulated expression of a set of GCC box-containing genes (Wu et al., 2002). In contrast, we found that silencing of *SlERF.A3* (*Pti4*) significantly suppressed growth of the pTRV2-SlERF.A3-infitlrated plants (**Figures 2B,C**), suggesting that a proper expression level of *SlERF.A3* (*Pti4*) is intrinsically required for at least vegetative growth in tomato.

It was demonstrated that plant ERF proteins could function as transcriptional activators or repressors (Fujimoto et al., 2000). However, several members of the B3 group of plant ERF family, including Arabidopsis ERF1 (AtERF92), AtERF15 (AtERF93), ORA59 (AtERF94), and ATERF14 (AtERF97) and tomato Pti4/5, TERF1, and TSRF1, have been shown to function as transcriptional activators (Zhou et al., 1997; Fujimoto et al., 2000; Gu et al., 2002; Wu et al., 2002; Huang et al., 2004; Zhang et al., 2007; Zarei et al., 2011). Recent biochemical studies showed that 12 members of the tomato B3 group, including SlERF.A1, SlERF.B4, and SlERF.C3, could bind to synthetic GCC promoter and acted as transcriptional activators, although the strength of transcriptional activity for members from different subgroups varied (Pirrello et al., 2012). In our study, we found that SlERF.A1, SlERF.A3, SlERF.B4, and SlERF.C3 were transcriptional activators and were localized in nucleus (**Figure 9**). It was found that, when overexpressed, several members in B3 group of the plant ERF family including tomato Pti4 and Arabidopsis ERF5 and ERF6 could upregulate expression of a large set of GCC box-containing genes (Wu et al., 2002; Moffat et al., 2012) and that the Arabidopsis ORA59 could directly bind to two functionally equivalent GCC boxes in the promoter of *PDF1.2* to enable its responsiveness to activation of the JA/ET signaling pathway (Zarei et al., 2011).

In this study, we took advantage of the simply and fast VIGS approach (Liu et al., 2002) to knockdown endogenous expression of individual member in the B3 group of the tomato ERF family for investigating their involvement in disease resistance against *B. cinerea*. However, functional redundancy is a common phenomenon among plant ERF genes with high levels of sequence similarity/identity, leading to a difficulty to characterize the requirement of members of plant ERF family in disease resistance against pathogens when use knockout or knockdown mutants. For example, neither of *erf5* nor *erf6* mutant plants displayed altered resistance to *B. cinerea*, while the *erf5 erf6* double mutant showed a significant increase in susceptibility to *B. cinerea*, demonstrating that ERF5 and ERF6, two members of IXb subgroup of B3 group of the Arabidopsis ERF family, play redundant roles in disease resistance against *B. cinerea* (Moffat et al., 2012). However, overexpression of either *ERF5* or *ERF6* led to increased disease resistance against *B. cinerea* (Moffat et al., 2012). In this regard, it is possible that we might miss discovery of other members in B3 group of the tomato ERF family that have function in disease resistance against *B. cinerea*. This is supported in part by the facts that silencing of *SlERF.C4* (*TSRF1*) or *SlERF.C6* (*Pti5*), which were previously shown to play important roles in disease resistance in overexpressing transgenic plants (He et al., 2001; Zhang et al., 2004b, 2007), did not affect disease resistance against *B. cinerea* (**Figure 3**). Although silencing of a specific ERF gene such as *SlERF.A1, SlERF.B4*, or *SlERF.C3* did not affect the expression of closely related ERF genes (**Figure 1B**), further detailed studies with consideration of functional redundancy among different members should be helpful in understanding the biological function of the B3 group members in tomato ERF family.

## Conclusion

Our VIGS-based functional analyses demonstrate that, SlERF.A1, SlERF.B4, and SlERF.C3, three previously uncharacterized members in the B3 group of the ERF family, and SlERF.A3, a previously identified ERF with function in immunity to *Pst* DC3000, are required for the resistance against *B. cinerea*. SlERF.A3 but not SlERF.A1, SlERF.B4 or SlERF.C3 is required for the resistance against *Pst* DC3000 in tomato. However, how SlERF.A1, SlERF.A3, SlERF.B4, and SlERF.C3 regulate tomato immunity against *B. cinerea* is still an open question. Currently, generation of stable transgenic tomato lines with overexpression and RNAi-mediated suppression of these defense-related ERF genes is undergoing in our lab. Once such transgenic lines are available, comparative RNA-seq and ChIP-seq analyses of transgenic plants with or without infection of *B. cinerea* will not only lead to the identification of SlERF.A1-, SlERF.A3-, SlERF.B4-, and SlERF.C3-dependent differentially expressed regulon, but also provide information on the SlERF.A1, SlERF.A3, SlERF.B4, and SlERF.C3 binding sites at genome-wide level. Data from these analyses will definitely be helpful to characterize the direct target genes, putative pathways and transcriptional network that are regulated by SlERF.A1, SlERF.A3, SlERF.B4, and SlERF.C3 during immune response against *B. cinerea*.

## Author Contributions

FS and ZO designed the experiments. ZO, SL, LhH, YH, XL, LH, YZ, HZ, and DL carried out most of the experiments. FS and ZO drafted and revised the manuscript. All authors read and approved the final manuscript.

## Conflict of Interest Statement

The authors declare that the research was conducted in the absence of any commercial or financial relationships that could be construed as a potential conflict of interest.
