# Supplementary material for: Tomato SlERF.A1, SlERF.B4, SlERF.C3 and SlERF.A3, Members of B3 Group of ERF Family, Are Required for Resistance to Botrytis cinerea
Source: Front Plant Sci. 2016 Dec 27;7:1964. doi: 10.3389/fpls.2016.01964 (PMC5187353; doi:10.3389/fpls.2016.01964)

**Supplementary Materials**

**Tomato SlERF.A1, SlERF.B4, SlERF.C3 and SlERF.A3, Members of B3 Group of ERF Family, Are Required for Resistance to *Botrytis cinerea***

***Zhigang Ouyang^1,2^, Lihong Huang^1^, Yongbo Hong^1^, Xiaohui Li^1^, Lei Huang^1^, Yafen Zhang^1^, Huijuan Zhang^1^, Dayong Li^1^, Fengming Song^1^***

*^1^National Key Laboratory for Rice Biology, Institute of Biotechnology, Zhejiang University, Hangzhou 310058, China; ^2^National Navel Orange Engineering Research Center, College of Life and Environmental Sciences, Gannan Normal University, China.*

**Supplementary File 1:** Sequences used as VIGS fragments for the B3 group of the tomato ERF genes

**>*SlERF.A1* (Solyc08g078180): 320 bp**

CTCGAATCAATTAGAAGATACTTACTTGAAGATTGGGAAGCTCCATTAACGAGCTCTGAAAACTCAACATCCTCAGAGTTCAGCCGGAGCAACAGCATTGAATCCAATATGTTTAGTAATTCATTTGATTATACACCTGAAATTTTTCAAAATGATATTCTTAATGAAGGATTTGGATTTGGATTTGAATTCGAGACTTCTGATTTTATAATCCCTAAATTAGAGTCACAAATGTCAATCGAATCACCTGAAATGTGGAATTTACCGGAATTTGTGGCTCCATTAGAGACGGCGGCGGAGGTGAAAGTTGAAACACCGGT

**>*SlERF.A2* (Solyc03g093610): 295 bp**

CAACTTCCCACTTCTACTGAGTTAACTTTTTTTCCGGCAGAATTCCCGGTGTATTGCCGGAGTTCAAGTTTCAGTAGTCTCATGCCATGTTTAACCGAATCATGGGGTGACTTGCCGTTAAAAGTTAACGATTCCGAAGATATGGTAATTTATGGGTTTCTACAAGACGCTTTTAGTATCGGATGGACGCCGTCAAATTTAACGTCCGAGGAAGTGAAACTCGAGCCGAGGGAGGAGATTGAGCCAGCTATGAGTACTTCTGTTTCTCCGCCGACAGTGGCTCCAGCGGCTTTGC

**>*SlERF-A3* (Solyc05g052050): 316 bp**

TCAACAGTTACCACCGACGAACTTCCCGGTAGATTTTCCGGTGTATCGCCGGAATTCAAGCTTCAGTCGTCTAATTCCCTGTTTAACTGAAAAATGGGGAGATTTACCACTAAAAGTCGATGATTCCGAAGATATGGTAATTTACGGTCTATTAAAAGACGCTCTAAGCGTCGGATGGTCGCCGTTTAATTTCACCGCCGGCGAAGTAAAATCGGAGCCGAGAGAAGAAATTGAATCGTCGCTTGAATTTTCACCTTCTCCGGCGGAGACCACGGCAGCTCCGGCGGCTGAAACACCGAAAGGAAGACATTATAGA

**>*SlERF.B1* (Solyc05g052040): 328 bp**

TCTTCACTAGAAATGATAAGACAACATCTTCTTGATGATGTTGTTTTCATGGAAACTTGTTCTTCTTCTTCTTCTTCTTCATTAGAAACAACAAGTAGTACACTTTATTCTCAAACCTCATCGAATTCGGAATCTTTAGAATCATTAACCTCTGAGATCAAACTTGAAAGCAATTTCTCTGTTTATCCTGATTTCATCAATACACCTCAAAGTTCAAATCTTGAATCTGTCTCTCGTTTTTTCGATAACTCAACTATTGAATTCCAAGCTAAACCCCAAAAGAAAAGAAGTTTCAATGATCGAAAACCTTCGTTAAACATTTCGATTC

**>*SlERF.B2* (Solyc03g093560): 271 bp**

ATGGGTTCTCCACAAGAGACTTGTACTTCACTTGATTTGATTAGGCAACATCTTTTTGATGAATCTCTGGACCAGACTTGTTTCTCTTTTGAAACAACTCAAACTTCAAATCTTGATGACATCGCAAGCTTCTTTAATGCTACTTCAAAAACAGAGTATGATGGTTTTTTCGAATTTGAGGCAAAACGACATGTTATCCATTCAAATTCTCCGAAACAGAGTAACTTGAGAGAACGGAAGCCATCTCTGAACGTAGCAATACCGGCGAAGC

**>*SlERF.B3* (Solyc05g052030): 305 bp**

TGACGAAACAAGATGAAGGATTAACATTAGAACTCATACGACAACATCTCCTCGAAGATTTCACAACTACAGAATCATTCATCGACAGTCTCAATTCTTGTTTTTCCGATCACATCTCCTCCTCCGATGACATCTCCCCTGTTTTCACTTCAGTAAAAACAGAGCCATCTACATCCAATTCCCTCTCAGATTCACCCAATTCCTCATACCCAAATGAACCCAACTCCCCAATTTCCCGTTACTTCAATCTCCGCTCCGATTTCCCTGAATTCAAAATCGATTCAGATACCATCCTCAGTCCAGTT

**>*SlERF.B4* (Solyc03g093540): 293 bp**

窗体顶端

TCACAAGAGATTTATACTTCACTTGATTCCATTAGGGAACATCTTCTTGATGATGATGTTACTTTTATGGAATATTACTGCTCTAAATCTTGTTTCTCTTTTCAAACTTCAAATCTTGACCATACCTCAAAAACAGAGTATGATGGTTTTTTCGAATTTGAGGCAAAACCACATGTTATCAGTTCAAATTCCCCGAAACAGAGTAACTTGAGAGAACGGAAGCCATCTCTGAACATTGCGATACCCGCGAAGCCAGTTGTTGTTGTAGAGAACGTTGAGAGTGAGAAGAAGCA

**>*SlERF.B5* (Solyc03g093550): 276 bp**

GCTCTCCACAAGAGATTTATACTTCACTTGATTCCATTAGGGAACATCTTCTTGATGATGATGTTGCTTTCATGGAATATTACTGCTCTAAATCTTGTTTCTCTTTTCAAACTTCAAATCTTGATCATACCTCAAAAACAGAGTACGATGGTTTTTTCAAATTTGAGGCAAAACCACATGTTATCAGTTCAAATTCTCCGAAACAGAGTAACTTGAGAGAACGGAAGCCATCTCTGAACATTGCGATACCCGCGAAGCCAATTGTTGTTGTAGAGA

**>*SlERF.B6* (Solyc01g090300): 246 bp**

ATTCCATCTTGTTAATGATTCCGATTTTTCTCAAATCCTTTCGATGTTCGATCCGATCAATATTAGTCATGCAGATATTATTAATAGTCCGAATTCGAGTTACGGTAGCTCTACATCAGCAGCAGAGATTAGTTGGGGAGATATGATAACTAATATTGATAGTCCGTGGCAATGTATCGATAAATTGGAGCACGAAGAGGCACCAAAAGAGGAGCCTTTAGTGGCACGTGGTGTGCACGCGCCTGG

**>*SlERF.B7* (Solyc01g090310): 251 bp**

TGAATCCATCTGATTTTTCTCTTCTTCAATCTATACAACATCATCTTCTTAATGATTCTGATTTTCCAAATATATTTTCAGCTATTGATTCCAATAATACCCCTACTGATTTTACACAAAATAATTTCGATTACGGGGAATTAACACCATTAATAAACTCTAGTACTACACTACAAGCCAACGAAAAATCCGAGGTTGAAGAGTCCGAGACAGTGGTGGCAAGTGTGAAACACGCACCAAAAGATTGGAAG

**>*SlERF.B13* (Solyc08g078190): 326 bp**

AGTAGTAAGTGTAGTGATACTGTGACGGAGAGTTGTGATTCTGAATCTGCTAGTTCTGGTTCTCTTCCTTTTGACTTTTTCTTTGATATTGAAGCTGATTTGTTTCAATTTGGCGCTGGAGTTTCTAATTCCAGCTCCGAATCAGCTAATTCGGTGTATAATCGAACAGATTTTGTTAAAATTGAATCGGAGCCGTCGATTTCAAGCTCTGATTATGAGGAGAATCAGAGTAAACGCTTTCAGTTTAAATCGGAACCTCAAGTATTCATCGATCTTACTTCACCGAAATCAAGGAAATTGTGTAGCGCTAGTGATAGGAAACCTTC

**>*SlERF.C1* (Solyc05g051200): 302 bp**

CAAGCCCACTAGAGATAGATACTTCATTTTCACATTCCAATTTGTTGTTTTTGGAAGATGAATCATCATGGAGTAATACTCATGATCCATTTGTTGATATTGATGAATATCTACCAATAATTATACCATGTAATGATGAAGAAATAGTAGTAGAATCCTCAAACACTAGTACTACAACAACAACAACAACAACATCAAAAGTAGCAAGTATCCAAAATATTCATCATGATCAAGAAGAGGTAACATCCATAGAGAAAAAACATGAAGATGATCAAGAAAAACATTATATTGGAGTTAGAAAG

**>*SlERF.C2* (Solyc04g014530): 301 bp**

TCATCCCCTAAAACTCAATATCCAAATTTCAATTTCTTCCAAGATCAATCATCATTACCATGGAATGATCAACATTTCTTAGATGAATATTTGACTAACATCGACCAAAACAACGATCATTCTCTACCAGGAAGTACTTGTTCATTCTTAACCTCGAAAGAAAGTTATAGACGGGAAGTTTCCTCCTCCAACCTACATCAATTACCAAGAAGTTGGTCATCCTCAAACGATACGAATTCCTCTAAAGAAAGCAATAATCGTCATGAAATCGAAGAGGTCACGTCTCATCACCATGATAAGA

**>*SlERF.C3* (Solyc09g066360): 271 bp**

TCTTTTAGTCATTGATTGAGCTTCAAACTTCCAAATAAGAATAGTCCAATCATATAGTAAATCAAATGGATTATTCATCTCGGGATGATCTTCTTTTTCACTATAATTCACTTCCATTTAACGTTAACGATACACAAGACATGTTACTTTATAATCTTGTTGCTGAAGGATCATCGCAAGAAACAGTGAATTCGTCGTCTAGCTATGGAATAAAGGAAGAGGAAGTGACCTCATATGAAGAAGAAAGAAAAGATAAGAATTACAGAGGTGT

**>*SlERF.C4* (Solyc09g089930): 264 bp**

GATTCTTCTTCTTCTTCATCTCAATTCTTCTACTCAATGAATTCTGATTTAAATTCATCAGATTCTTCATACGAATGGTCCAATTTCAACACACAATCTTATCTCCCTTTCAACGTGAACGACTCCGAAGAGATGCTTCTCTTCGGAGTTCTTAACGCTGCTCATGAAGAAACAACATCCGAAACAGTCACATCGCATCGCGTTAAAGAAGAAGAAGTTACCTCAGAATCCGAGGTTATTGAAGCAATACCGGCGAAGGAGAAG

**>*SlERF-C05* (Solyc02g077360): 264 bp**

TCCAAGAATGATCAAAATTCTCAAAAGGTGACAAATGAATATTGTTGTGAACAATATTGGGAACAACTTCCAATTCTTGAAAGATTACCAAGCTTAGGAAGATGGATATCAATGGGAGCTGAAACTTGGGAAGATATTCTCAATGGAATTATTATTCCTTCTCATAACAACGAAAATTCAAACGACGAATCAACATGCAAAGATGTTGTCAACGTTGAGAAGAAGGAGGAGAAGAAGAAGATGGTGCATTATAGAGGGGTAAGA

**>*SlERF.C6* (Solyc02g077370): 257 bp**

ATGGTTCCAACTCCTCAAAGTGATTTACCTCTTAATGAGAATGACTCACAAGAGATGGTATTATATGAAGTTCTTAATGAAGCTAATGCTCTAAATATTCCTTATTTACCCCAACGAAATCAATTACTCCCTAGAAATAATATTCTTCGTCCATTACAGTGCATAGGCAAGAAATACAGAGGAGTACGACGTCGTCCGTGGGGGAAATACGCTGCGGAAATTCGCGATTCGGCTAGACATGGTGCGAGAGTATGGCT

**>*SlERF.C11* (Solyc09g089910): 309 bp**

TGGAATTAAAATTAAACATGGAACAAGATCCTAGCAATGAAAGAAGAACTAATGGTGGTGGCGGAGAGATAAAATATCGAGGCGTACGTAGGCGTCCGTGGGGTAAATTTGCAGCGGAGATACGTGACTCAGCACGACAAGGGGCACGTGTATGGCTAGGGACATTTAATACTGCGGAAGAAGCAGCAAGAGCTTATGATAGGGCAGCTTATTCAATGAGGGGACATTTAGCTATACTAAATTTTCCTGAGGAGTATAATTTACCTAGTAGCTCTTCACATTTTTATAGTGCCGGTTCTTATTCTTCAT

**Supplementary Table S1 Primers used in this study**

| Primer name | Primer 5’-3’ | Size (bp) |
| --- | --- | --- |
| ***Subcelluar localization*** | |  |
| SlERF.A1-1F | GC TCTAGA ATGATTTTTCAGTACTCGAATC (*Xba*I) | 678 |
| SlERF.A1-1R | TCC CCCGGG TTATTGCTTTGTTCCACGAGCA (*Sma*I) |
| SlERF.B4-1F | GC TCTAGA ATGGGCTCTTCACAAGAGAT (*Xba*I) | 687 |
| SlERF.B4-1R | TCC CCCGGG TCAAAGTAAATCAAAAATGCCTTT (*Sma*I) |
| SlERF.C3-1F | GC TCTAGA ATGGATTATTCATCTCGGGA (*Xba*I) | 579 |
| SlERF.C3-1R | TCC CCCGGG CCAATTTGTGATACTTTCTGAA (*Sma*I) |
| SlERF.A3-1F | GC TCTAGA ATGGATCAACAGTTACCACC | 705 |
| SlERF.A3-1R | TCC CCCGGG TTAAATGACCAATAGTTGAT |
| ***Transcription activation*** | |  |
| SlERF.A1-2F | GC CCCGGG ATGATTTTTCAGTACTCGAATC (*Sma*I) | 678 |
| SlERF.A1-2R | GCGCTGCAG TTATTGCTTTGTTCCACGAGCA (*Pst*I) |
| SlERF.B4-2F | GC CCCGGG ATGGGCTCTTCACAAGAGAT (*Sma*I) | 687 |
| SlERF.B4-2R | GCGCTGCAG TCAAAGTAAATCAAAAATGCCTTT (*Pst*I) |
| SlERF.C3-2F | GC CCCGGG ATGGATTATTCATCTCGGGA (*Sma*I) | 579 |
| SlERF.C3-2R | GCGCTGCAG CCAATTTGTGATACTTTCTGAA (*Pst*I) |
| SlERF.A3-1F | GC CCCGGG ATGGATCAACAGTTACCACC | 705 |
| SlERF.A3-1R | GCGCTGCAG TTAAATGACCAATAGTTGAT |
| ***qRT-PCR*** | |  |
| SlERF.A1-3F | CGTGGAACAAAGCAATAAGTCC | 190 |
| SlERF.A1-3R | AGCCAAACTCGTGCTCCATT |
| SlERF.A2-3F | TGAATCGGCTAGTTCATCGG | 159 |
| SlERF.A2-3R | TCGCCAACTGGAAATTGTTC |
| SlERF.A3-3F | ATCAACAGTTACCACCGACGAA | 193 |
| SlERF.A3-3R | AAATTAAACGGCGACCATCC |
| SlERF.B1-3F | CTTCAATTTGGGATTGTGGA | 184 |
| SlERF.B1-3R | AGTGGATCGTGATGGCAGTA |
| SlERF.B2-3F | CAAGAAGGAAAGGAAAATAGAAGA | 140 |
| SlERF.B2-3R | TAACAAGCTGAGATAATGGTGACA |
| SlERF.B3-3F | TCAAAATACTGCAAAATGACGA | 170 |
| SlERF.B3-3R | ACTGAAGTGAAAACAGGGGAGA |
| SlERF.B4-3F | CCATTAGGGAACATCTTCTTGA | 181 |
| SlERF.B4-3R | AAGTTACTCTGTTTCGGGGAAT |
| SlERF.B5-3F | CCATTAGGGAACATCTTCTTGA | 173 |
| SlERF.B5-3R | CTGTTTCGGAGAATTTGAACTG |
| SlERF.B6-3F | TTAGTTATCATATCTCCCCAAC | 132 |
| SlERF.B6-3R | CGATTTTTCTCAAATCCTTTCG |
| SlERF.B7-3F | TTGGTCATAAGCCAATGCTG | 193 |
| SlERF.B7-3R | CGAGGTTGAAGAGTCCGAGA |
| SlERF.B13-3F | CGATTGTTTCGGTTCCTGTG | 100 |
| SlERF.B13-3R | ATCTCCGCCGCGTACTTTCC |
| SlERF.C1-3F | ATGATCCATTTGTTGATATTGATG | 128 |
| SlERF.C1-3R | ACTTGCTACTTTTGATGTTGTTGT |
| SlERF.C2-3F | AGAATGAAAACAAAATAGAAGCAA | 186 |
| SlERF.C2-3R | TTAAAGGCAACAAATAAAATGAGA |
| SlERF.C3-3F | AAGATTTGGGTGCTGAGTATTT | 169 |
| SlERF.C3-3R | CCCAATTTTCCCTCCGTTAA |
| SlERF.C4-3F | GAAACAGTCACATCGCATCG | 107 |
| SlERF.C4-3R | CGCCTCCTAACACCTCGGTA |
| SlERF.C5-3F | GTACTAAATGTCCCTAGCCAAACC | 180 |
| SlERF.C5-3R | CAACGAAAATTCAAACGACGAATC |
| SlERF.C6-3F | TGAATCAAGATGTTAAAACAGAGC | 168 |
| SlERF.C6-3R | AAGGAAAATAAGAGAAGAGGCAAG |
| SlERF.C11-3F | TAGTGCCGGTTCTTATTCTTCA | 106 |
| SlERF.C11-3R | TTCCTCCAACAACTTATCATCCA |
| ***VIGS*** | |  |
| SlERF.A1-4F | GAT GGATCC CTCGAATCAATTAGAAGATACT (*Bam*HI) | 320 |
| SlERF.A1-4R | TCC CCCGGG ACCGGTGTTTCAACTTTCACCT (*Sma*I) |
| SlERF.A2-4F | GAT GGATCC CAACTTCCCACTTCTACTGAGT (*BamH*I) | 295 |
| SlERF.A2-4R | TCC CCCGGG GCAAAGCCGCTGGAGCCACTGT (*Sma*I) |
| SlERF.A3-4F | GAT GGATCC TCAACAGTTACCACCGACGAACT (*BamH*I) | 316 |
| SlERF.A3-4R | TCC CCCGGG TCTATAATGTCTTCCTTTCGGT (*Sma*I) |
| SlERF.B1-4F | GAT GGATCC TCTTCACTAGAAATGATAAGAC (*BamH*I) | 328 |
| SlERF.B1-4R | TCC CCCGGG GAATCGAAATGTTTAACGAAGGT (*Sma*I) |
| SlERF.B2-3F | GAT GGATCC GTTCTCCACAAGAGACTTGTACT (*BamH*I) | 267 |
| SlERF.B2-4R | TCC CCCGGG GCTTCGCCGGTATTGCTACGT (*Sma*I) |
| SlERF.B3-4F | GAT GGATCC TGACGAAACAAGATGAAGGAT (*BamH*I) | 305 |
| SlERF.B3-4R | TCC CCCGGG AACTGGACTGAGGATGGTATCT (*Sma*I) |
| SlERF.B4-4F | GAT GGATCC TCACAAGAGATTTATACTTCACT (*BamH*I) | 293 |
| SlERF.B4-4R | TCC CCCGGG TGCTTCTTCTCACTCTCAACGT (*Sma*I) |
| SlERF.B5-4F | GAT GGATCC GCTCTCCACAAGAGATTTATACT (*BamH*I) | 273 |
| SlERF.B5-4R | TCC CCCGGG TCTCTACAACAACAATTGGCT (*Sma*I) |
| SlERF.B6-4F | GAT GGATCC ATTCCATCTTGTTAATGATTC (*BamH*I) | 246 |
| SlERF.B6-4R | TCC CCCGGG CCAGGCGCGTGCACACCACGT (*Sma*I) |
| SlERF.B7-4F | GAT GGATCC TGAATCCATCTGATTTTTCTCT (*BamH*I) | 251 |
| SlERF.B7-4R | TCC CCCGGG CTTCCAATCTTTTGGTGCGTGT (*SmaI*) |
| SlERF.B13-4F | GAT GGATCC AGTAGTAAGTGTAGTGATACT (*BamH*I) | 326 |
| SlERF.B13-4R | TCC CCCGGG GAAGGTTTCCTATCACTAGCGCT (*Sma*I) |
| SlERF.C1-4F | GAT GGATCC CAAGCCCACTAGAGATAGATACT (*BamH*I) | 302 |
| SlERF.C1-4R | TCC CCCGGG CTTTCTAACTCCAATATAATGT (*Sma*I) |
| SlERF.C2-4F | GAT GGATCC TCATCCCCTAAAACTCAATATC (*BamH*I) | 301 |
| SlERF.C2-4R | TCC CCCGGG TCTTATCATGGTGATGAGACGT (*Sma*I) |
| SlERF.C3-4F | GAT GGATCC TCTTTTAGTCATTGATTGAGCT (*BamH*I) | 271 |
| SlERF.C3-4R | TCC CCCGGG ACACCTCTGTAATTCTTATCT (*Sma*I) |
| SlERF.C4-4F | GAT GGATCC GATTCTTCTTCTTCTTCATCT (*BamH*I) | 264 |
| SlERF.C4-4R | TCC CCCGGG CTTCTCCTTCGCCGGTATTGCT (*Sma*I) |
| SlERF.C5-4F | GAT GGATCC TCCAAGAATGATCAAAATTCT (*BamH*I) | 264 |
| SlERF.C5-4R | TCC CCCGGG TCTTACCCCTCTATAATGCAC (*Sma*I) |
| SlERF.C6-4F | GAT GGATCC ATGGTTCCAACTCCTCAAAGT (*BamH*I) | 257 |
| SlERF.C6-4R | TCC CCCGGG AGCCATACTCTCGCACCATGT (*Sma*I) |
| SlERF.C11-4F | GAT GGATCC TGGAATTAAAATTAAACATGGA (*BamH*I) | 309 |
| SlERF.C11-4R | TCC CCCGGG ATGAAGAATAAGAACCGGCACT (*Sma*I) |
| ***Defense genes*** | |  |
| SlActin-RT-F | CCAGGTATTGCTGATAGAATGAG | 113 |
| SlActin-RT-R | GAGCCTCCAATCCAGACAC |
| SlPin2-RT-F | AATTTATCCCACCGGATGTACC | 102 |
| SlPin2-RT-R | GGTTCATCACTCTCTCCTTCAC |
| SlLapA1-RT-F | CTGGGAACTGGACCTGAAATAG | 83 |
| SlLapA1-RT-R | ACGAGCTCTCTACCGAGTATAA |
| SlPR1a-RT-F | CGTAAGGCGGCTCAATAAGT | 106 |
| SlPR1a-RT-R | GATTGCATGTCGTGTGATTAAGG |
| SlPR-P2-RT-F | CGATCTAAATTGATTTCATAGTACG | 116 |
| SlPR-P2-RT-R | TCGTGAAGGATATACAAAATACA |

**Supplementary Figure S1:** Phylogenetic tree of cDNA sequences of the tomato B3 group ERF genes.

Phylogenetic tree was constructed using neighbor-joining method and evaluated by the interior branch test method using MEGA 6.0 software with 1000 bootstrap replicates.


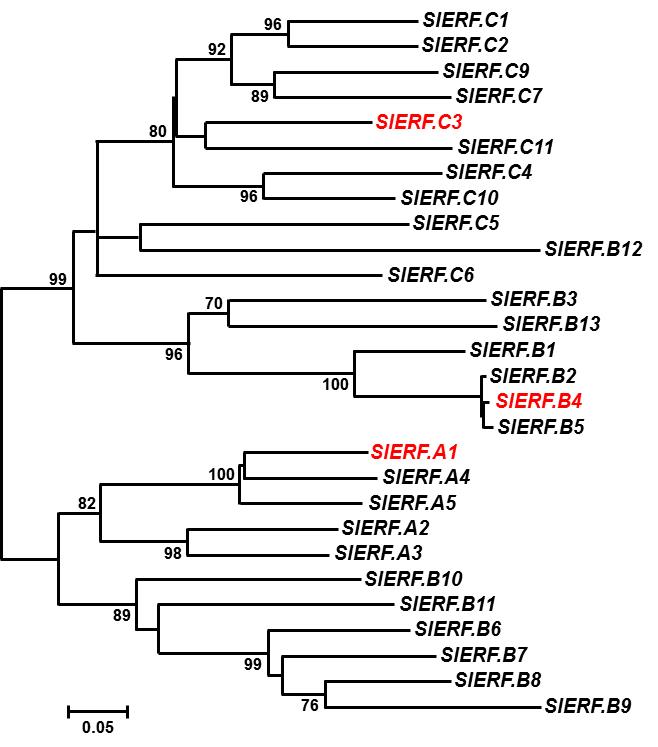

Supplement: Supplementary file 1 [file Table_1.DOC]
